# Untargeted Metabolomic Study of Lung Cancer Patients after Surgery with Curative Intent

**DOI:** 10.1021/acs.jproteome.3c00356

**Published:** 2023-10-16

**Authors:** Saida Sanchez-Espirilla, Antonio Pereira-Vega, Belén Callejón-Leblic, Isabel Díaz-Olivares, Rafael Santana, Carolina Gotera Rivera, José Luis Gómez-Ariza, José Luis López-Campos, Ana Isabel Blanco-Orozco, Luis Seijo, María Rodríguez, Luis Alejandro Padrón Fraysse, Ángeles Herrera-Chilla, Germán Peces-Barba, Tamara García Barrera

**Affiliations:** † Department of Chemistry, Research Center for Natural Resources, Health and the Environment (RENSMA), Faculty of Experimental Sciences, University of Huelva, Campus El Carmen, Fuerzas Armadas Ave., 21007 Huelva, Spain; ‡ Department of Chemistry, Faculty of Sciences, National University of San Antonio Abad of Cusco, Av. de La Cultura, 773 Cusco, Peru; § Pneumology Area of the Juan Ramón Jiménez Hospital, Ronda Norte, s/n, 21005 Huelva, Spain; ∥ IIS Jiménez Díaz Foundation, ISCIII-CIBERES, Reyes Católicos Ave., 28040 Madrid, Spain; ⊥ Medical-Surgical Unit of Respiratory Diseases, Institute of Biomedicine of Seville (IBiS), Antonio Maura Montaner, 41013 Seville, Spain; # Virgen del Rocío University Hospital/University of Seville, Manuel Siurot, s/n, 41013 Sevilla, Spain; ∇ Center for Biomedical Research in Respiratory Diseases Network (CIBERES), Carlos III Health Institute, Monforte de Lemos Ave., 28029 Madrid, Spain; ○ University Clinic of Navarra, Marquesado de Santa Marta Street, 1, 28027 Madrid, Spain

**Keywords:** metabolomics, mass spectrometry, hydrophilic interaction, lung cancer, serum, surgery, ultra-high performance liquid chromatography

## Abstract

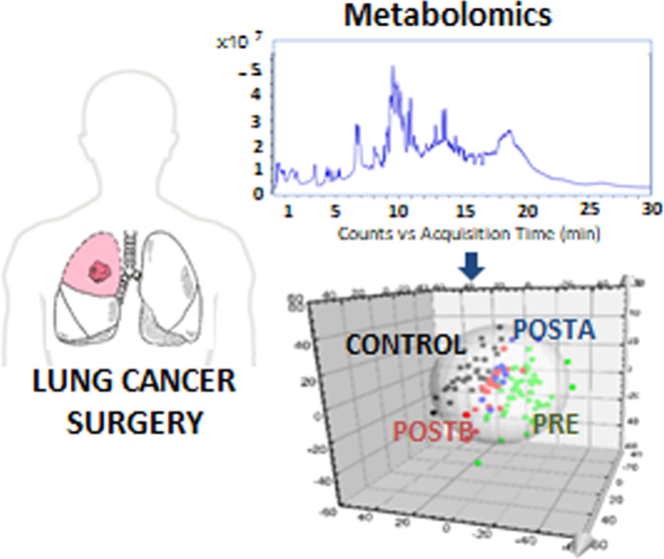

Lung cancer (LC) is a leading cause of mortality, claiming more than 1.8 million deaths per year worldwide. Surgery is one of the most effective treatments when the disease is in its early stages. The study of metabolic alterations after surgical intervention with curative intent could be used to assess the response to treatment or the detection of cancer recurrence. In this study, we have evaluated the metabolomic profile of serum samples (*n* = 110) from preoperative (PRE) and postoperative (POST) LC patients collected at two different time points (1 month, A; 3–6 months, B) with respect to healthy people. An untargeted metabolomic platform based on reversed phase (RP) and hydrophilic interaction chromatography (HILIC), using ultra-high performance liquid chromatography (UHPLC) and mass spectrometry (MS), was applied (MassIVE ID MSV000092213). Twenty-two altered metabolites were annotated by comparing all the different studied groups. DG(14,0/22:1), stearamide, proline, and E,e-carotene-3,3′-dione were found altered in PRE, and their levels returned to those of a baseline control group 3–6 months after surgery. Furthermore, 3-galactosyllactose levels remained altered after intervention in some patients. This study provides unique insights into the metabolic profiles of LC patients after surgery at two different time points by combining complementary analytical methods.

## Introduction

Lung cancer (LC) is the second most detected cancer in the world, representing the first and third leading cause of death in men and women, respectively.^1^ Five-year LC survival rates are as low as 4% when patients are diagnosed in the advanced stages of the disease, although this rate can increase up to 65% when the disease is detected in the early stages and treatments with curative intent are effective.^1^ Likewise, the most appropriate treatment for the early stages of non-small cell LC (NSCLC) (stages I and II) is surgery. In spite of surgical intervention, many NSCLC patients need chemotherapy, radiation, target therapy, immunotherapy, or some combination of these treatments to prevent a recurrence.^2^ Despite the fact that the survival rate increases with surgical treatment, life expectancy is limited by recurrence, so follow-up after surgery is the norm. In this sense, the identification of biomarkers that provide information on possible metabolic changes before or after surgery could improve prevention, early detection, and guide adjuvant or neoadjuvant therapy in order to avoid possible disease recurrence.

Metabolomics is considered a powerful approach for investigating the behavior of a wide number of metabolites in a large variety of biological samples, including serum. Most metabolomic studies of LC have analyzed biological samples from patients with LC and healthy people to establish metabolic differences between them and identify potential biomarkers for early diagnosis.^3−7^ However, few studies have investigated metabolic alterations in LC patients before and after surgery. Ahmed et al.^10^ analyzed serum and urine samples from preoperative and postoperative LC patients 4 months after surgery and observed an increase in the levels of lipid and carboxylic acids. Yang et al.^8^ also described alterations in lipids, fatty acids, and amino acids in preoperative and postoperative LC patients 7 days post-resection compared to a control group. Similarly, Chen et al.^9^ reported changes in many metabolites involved in lipid metabolism in LC patients before and after surgical intervention. The metabolomic studies in serum from preoperative and postoperative LC patients have been mainly carried out by using reverse phase (RP) liquid chromatography coupled to a quadrupole time-of-flight mass spectrometry analyzer (HPLC-QTOF-MS)^9,10^ or gas chromatography-mass spectrometry (GC-MS).^9^ However, the use of hydrophilic interaction ultra-high performance liquid chromatography (HILIC) in metabolomics has currently gained growing interest because it allows the determination of a wide number of polar metabolites. In this sense, only Yang et al. have employed this novel analytical technique, to study a limited number of LC patients undergoing surgical resection. Thus, the objective of this work was to determine and identify metabolites that can potentially indicate a good prognosis, failure of the intervention, or possible recurrence of LC after surgical intervention with curative intent using metabolomics.

## Methods

### Study Design

This study aims to analyze the variations in the global metabolomic profile of patients with NSCLC in the early stages who underwent surgery with curative intent. The patients in the study do not have another type of cancer and have not been treated with chemotherapy, radiotherapy, or immunotherapy. This is a prospective longitudinal study consisting of two phases: a blood sample collection phase before and after surgery (1 to 6 months after surgery); and a patient follow-up phase in which samples are collected every 3 months for 3 years after surgery, which is still in progress. Control blood samples were collected from healthy volunteers.

### Sample Collection

LC and control samples were collected at three different Spanish hospitals. Blood samples were obtained by venipuncture of the antecubital region after 8 h of fasting and collected in BD Vacutainer SST II tubes with a gel separator and Advance vacuum system. The samples were immediately cooled and protected from light for 30 min to allow for clot retraction. After centrifugation (2057*g* for 10 min), serum samples were aliquoted in Eppendorf tubes and frozen at −80 °C until analysis.

Samples were divided into 4 groups: a control group of healthy people (CONTROL, 35 samples), a group of preoperative NSCLC patients (PRE, 48 samples), and two groups of postoperative LC patients 1 month (POSTA, 15 samples) and 3–6 months after surgery (POSTB, 17 samples). Clinical data are shown in Table S1 in the Supporting Information.

### Reagents

All of the solvents used were of HPLC-grade. Methanol, ethanol, acetronitrile, and pyridine were purchased from Aldrich (Steinheim, Germany). Formic acid and ammonium formate were supplied by Merck (Darmstadt, Germany). Water was purified with a Milli-Q Gradient system (Millipore, Watford, UK). Palmitic acid-d31 used as the internal standard, was purchased from Aldrich (Steinheim, Germany).

### Sample Treatment

For RP analysis, the extraction of metabolites from serum samples was carried out by adding 400 μL of a mixture of methanol/ethanol (1:1 v/v) to 100 μL of serum into Eppendorf tubes. The samples were vortexed for 5 min at room temperature, followed by centrifugation at 2057*g* for 10 min at 4 °C to eliminate the sediment containing the protein fraction. The supernatant was transferred to another Eppendorf tube and dried in a fast vacuum system (Thermo Scientific Savant SPD111 V SpeedVac Concentrator) at 30 °C for 20 min. The resulting residue was reconstituted with 100 μL of a methanol/water (8:2 v/v) mixture for the analysis. After that, the remaining pellet was extracted twice with a mixture of acetonitrile/methanol (4:1 v/v) for the extraction of nonpolar metabolites, vortexed and centrifuged with the same previous conditions, and dried using a nitrogen stream. These extracts were reconstituted in 100 μL of acetonitrile/methanol (6:4 v/v) with 10 mM ammonium formate. Finally, for HILIC analysis, samples were extracted by the addition of 400 μL of methanol/water (4:1 v/v). Then, the samples were vortexed and centrifuged using the same previous conditions and dried in the fast vacuum system (Thermo Scientific Savant SPD111 V SpeedVac Concentrator) at 30 °C for 60 min. The extracts were reconstituted in 100 μL of methanol/water (4:1 v/v). The internal standard palmitic acid-d31 was added to the samples for quality control.

### Instrumentation: UHPLC-QTOF-MS

In order to achieve wide metabolic coverage, an RP and a HILIC coupled to UHPLC were combined. Chromatographic separations were carried out on an Agilent 1290 series coupled to an Agilent 6550 iFunnel Q-TOF-MS instrument equipped with a dual electrospray ionization (ESI) source operated in negative and positive mode (Agilent Technologies, Tokyo, Japan).

For the analysis by RP-UHPLC-QTOF-MS, water (phase A) and acetonitrile (phase B) with 0.1% of formic acid were used as mobile phases following gradient conditions from 5 to 100% of phase B with a total chromatogram time of 30 min. The chromatographic separation was carried out in a Zorbax C18, 1.5 μm, 30 mm × 2.1 mm I.D column (Agilent Technologies) in both ionizations, positive and negative modes.

For HILIC-UHPLC-QTOF-MS analysis, mobile phases were composed of 20 mM ammonium formate and 0.1% formic acid in water (phase A) and acetonitrile (phase B) with 0.1% formic acid. The gradient elution was set to 95% down to 45% of B with a total time of 15 min. The flow rate was set at 0.4 mL min^–1^. The chromatographic separation was carried out in Acquity BEH Amide, 1.7 μm, 100 mm × 2.1 mm ID column (Waters, Massachusetts, USA).

The reference masses used for the mass correction were *m*/*z* 121.0509 and *m*/*z* 922.0098 amu, which were constantly introduced into the system for both ionization modes (positive and negative). The mass range was monitored from 50 to 1100 amu. The QTOF parameters were set to 3 kV for the capillary voltage, 11 L min 1 at 350 °C for the drying gas flow rate, and 35 psi for the gas nebulizer. The fragmentor voltage was set to 175 V for both ionization modes. Samples were acquired in full scan mode (MS) for the primary untargeted analysis. Then, a list containing the most significant features was imported and analyzed in targeted MS/MS mode with MS/MS scan rate of 1 spectrum s^–1^ using the initial chromatographic conditions. Nitrogen was used as collision gas, and several collision voltages were fixed from 10 to 40 V for the fragmentation of compounds. Data were acquired at centroid mode using a scan rate of 1.0 spectra per second.

### Data Processing

For UHPLC-QTOF-MS, raw data processing was carried out with Agilent MassHunter Profinder B.10.0 software (Agilent Technologies). To extract the data, batch recursive feature extraction (RFE) for small molecules wizard from the software was applied. RFE performs two algorithms. First, the molecular feature extraction algorithm (MFE), including extraction, selection of ion species, and charge state, was used to find the features in the data set. Second, the initial features were aligned by the retention time (RT) and mass, creating a list of unique features through binning. Then, the RT and mass data pairs of the aligned and binning features were used as input criteria to more accurately find the features using the Find by Ion algorithm (FbI). Additional filters, such as scoring, integration, and peak filters, were also applied to the data set. Table S2 shows the parameters and filters used for the positive and negative modes. Moreover, Mass Profiler Professional B.10.0 (Agilent Technologies) was used for the normalization of the data set using the internal standard.

### Statistical Analysis

For UHPLC-QTOF-MS data processing, Mass Profiler Professional B.10.0 (Agilent Technologies) was used for the determination of the most relevant metabolites between groups. For both features determined by the RP and HILIC UHPLC-QTOF-MS methodologies, principal component analysis (PCA) and partial least-squares discriminant analysis (PLS-DA) were carried out in order to compare the serum metabolomic profiles obtained. PCA plots showed a good clustering of the QCs samples (Figure S1, Supporting Information), demonstrating the stability and reliability of the metabolomics approach. Table S3 (Supporting Information) reported the coefficients of variation (CV) of QCs that were used to select those compounds with values lower than 15%. The predictive and class separation parameters *R*^2^ and *Q*^2^ of all models built were supplied by the software (Table S4, Supporting Information). Before statistical analysis was performed, the data were submitted to Pareto scaling and logarithmic transformation.

To assess the specificity and sensitivity of the altered metabolites, the values of the areas under the curve (AUC) of the received operator characteristics (ROC) analysis were determined using Metaboanalyst 5.0. (https://www.metaboanalyst.ca/). AUC values higher than 0.75 were considered clinically useful in medicine.^11^

One-way ANOVA and the Tukey test for multiple comparisons were applied using STATISTICA 8.0 from StatSoft. Moreover, a Benjamini–Hochberg FDR correction was also applied to adjust the p-values. The level of statistical significance for all tests was set to *p* < 0.05.

### Annotation of Serum Metabolites

According to recommendations by the Metabolomics Standards Initiative (MSI), metabolites were identified to MSI Level 2.^12^ The Agilent Qualitative Analysis Workflow MassHunter B.08.00 software was used to annotate the compounds. For this purpose, the workflow “Compound Discovery” and the compound mining “Find by Molecular Features” from the software were applied to the data set. METLIN (http://metlin.scripps.edu) and HMDB (http://hmdb.ca) databases were consulted for the annotation of altered compounds, considering a score higher than 97%, which reflects how well the compound matches the mass, isotope pattern, and retention time of the target compound.

Moreover, MS-MS experiments were applied to samples in order to confirm the annotation of some compounds using a QTOF (6550 system, Agilent Technologies) with the same chromatographic conditions as those applied for the primary analysis. Ions were targeted by collision-induced dissociation fragmentation on the fly based on the previously determined accurate mass and retention time.

## Results

Metabolomic profiles of serum samples from CONTROL, PRE, POSTA, and POSTB groups were determined using both methodologies ESI(±)-RP-UHPLC-QTOF-MS and ESI(+)-HILIC-UHPLC-QTOF-MS. Figure S2 of the Supporting Information shows the characteristic metabolomic profiles of the different extracts of a human serum sample determined by ESI(±)-RP-UHPLC-QTOF-MS and ESI(+)-HILIC-UHPLC-QTOF-MS. Blanks were prepared using the same procedure as samples and analyzed at the beginning and end of the batch to ascertain the absence of contamination and artifacts during the UHPLC-QTOF-MS analysis.

**Figure 1:**
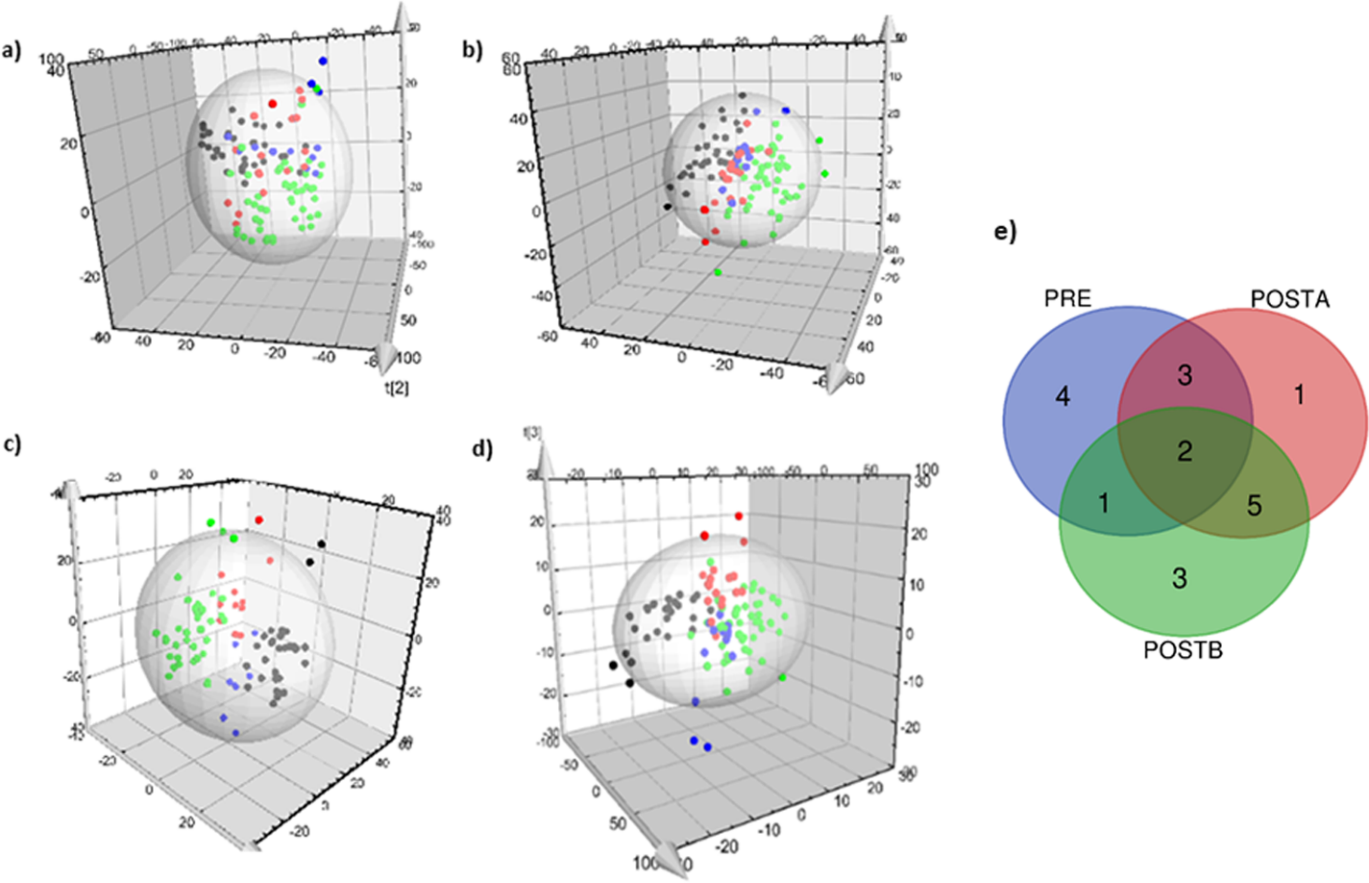
3D-PLS-DA scatter plot of (a) MeOH:H_2_O (8:1 v/v)extracts determined by ESI(+)-RP-UHPLC-QTOF-MS and (b) ESI(−)-RP-UHPLC-QTOF-MS; (c) ACN:MeOH (6:4 v/v) extracts determined by ESI(+)-RP-UHPLC-QTOF-MS; (d) MeOH:H_2_O (4:1 v/v) extracts determined by ESI(+)-HILIC-UHPLC-QTOF-MS; (e) Venn diagram with the common number of metabolites in the different studied groups. CONTROL: black dots; PRE: green dots; POSTA: blue dots; and POSTB: red dots.

PLS-DA showed good classifications between groups in the different organic and aqueous extracts analyzed by ESI (±)-RP-UHPLC-QTOF-MS and ESI(+)-HILIC-UHPLC-QTOF-MS (Figure 1a–d). The 3D-PLS-DAs built from any pairwise group comparison (Figures S3–S7, Supporting Information) are reported in the Supporting Information, showing good discrimination between groups.

Twenty-two altered metabolites (Table S5) were annotated combining HILIC-UHPLC-QTOF-MS (10 metabolites) and RP-UHPLC-ESI-(±)-QTOF-MS (12 metabolites). The metabolites were identified using the METLIN database using a score higher than 90%, and with MS/MS analysis to confirm the identity of the metabolites with the characteristic fragments (Table S6). The coefficient of variation of the abundance of altered metabolites to check the dispersion per group is shown in Supporting Information (Table S7). The levels of 9 metabolites were significantly altered (*p* < 0.05) in LC patients compared to the control group. These metabolites were stearamide (0.38-fold), DG(14:0/22:1) (2.06-fold), DG(16:0/24:1) (1.68-fold), 6,10,14-trimethyl-5,9,13-pentadecatrien-2-one (0.63-fold), E,e-carotene-3,3′-dione (1.79-fold), 3-galactosyllactose (1.45-fold), proline (0.63-fold), glucosylgalactosyl hydroxylysine (1.19-fold), 3-*b*-galactopyranosyl glucose (1.16-fold) and l-carnitine (1.16-fold).

Moreover, we observed significant postoperative changes in a total of 10 metabolites immediately after surgery in the POSTA group when compared to the control group, including 6-(2-carboxyethyl)-7-hydroxy-2,2-dimethyl-4-chromanone glucoside (5.36-fold), butyl ethyl malonate (4.34-fold), DG(14:0/22:1) (6.13-fold), 1-methylhistidine (0.86-fold), 3-galactosyllactose (1.59-fold), argininic acid (1.15-fold), cystine (1.21-fold), glucosylgalactosyl hydroxylysine (1.24-fold), l-carnitine (1.18-fold), *N*-(1-deoxy-1-fructosyl)leucine (0.91-fold), and proline (0.63-fold).

Long-term follow-up (POSTB), revealed 9 metabolites altered when compared to the control group including 6-(2-carboxyethyl)-7-hydroxy-2,2-dimethyl-4-chromanone glucoside (2.29-fold), butyl ethyl malonate (1.66-fold), choline (1.97-fold), DG(14:0/22:1) (1.29-fold), 1-methylhistidine (0.81-fold), 3-*b*-galactopyranosyl glucose (1.22-fold), 3-galactosyllactose (1.48-fold), argininic acid (1.14-fold), and cystine (1.29-fold).

Interestingly, glucosylgalactosyl hydroxylysine, l-carnitine, and proline were significantly altered before and immediately after surgery but returned to levels similar to the control group baseline after 6 months of surgery, while 3-*b*-galactopyranosyl glucose was altered before surgery and after 6 months of surgery, but not immediately after surgery. Finally, we found that 6,10,14-trimethyl-5,9,13-pentadecatrien-2-one, DG(16:0/24:1), E,e-carotene-3,3′-dione, and stearamide were significantly altered before but not after surgery. We also found significant differences in the abundance of 6-(2-carboxyethyl)-7-hydroxy-2,2-dimethyl-4-chromanone glucoside, butyl ethyl malonate, linalyl propianate, LysoPC(17:0), 3-galactosyllactose, and 3-*b*-galactopyranosyl glucose after surgery, but not before the intervention, suggesting metabolic changes related to the intervention itself. Figure 1d represents a Venn diagram showing the number of common and different metabolites in the studied groups. As we can see, two altered metabolites (3-galactosyllactose and DG (14:0/22:1)) were common in PRE, POSTA, and POSTB groups, 3 metabolites (glucosylgalactosyl hydroxylysine, proline, and l-carnitine) were common in PRE and POSTA groups, and 5 metabolites (1-methylhistidine, butyl ethyl malonate, cysteine 6-(2-Carboxyethyl)-7-hydroxy-2,2-dimethyl-4-chromanone glucoside, and argininic acid were common in POSTA and POSTB. The complete results for the Venn diagram are shown in Table S8 in Supporting Information.

**Figure 2:**
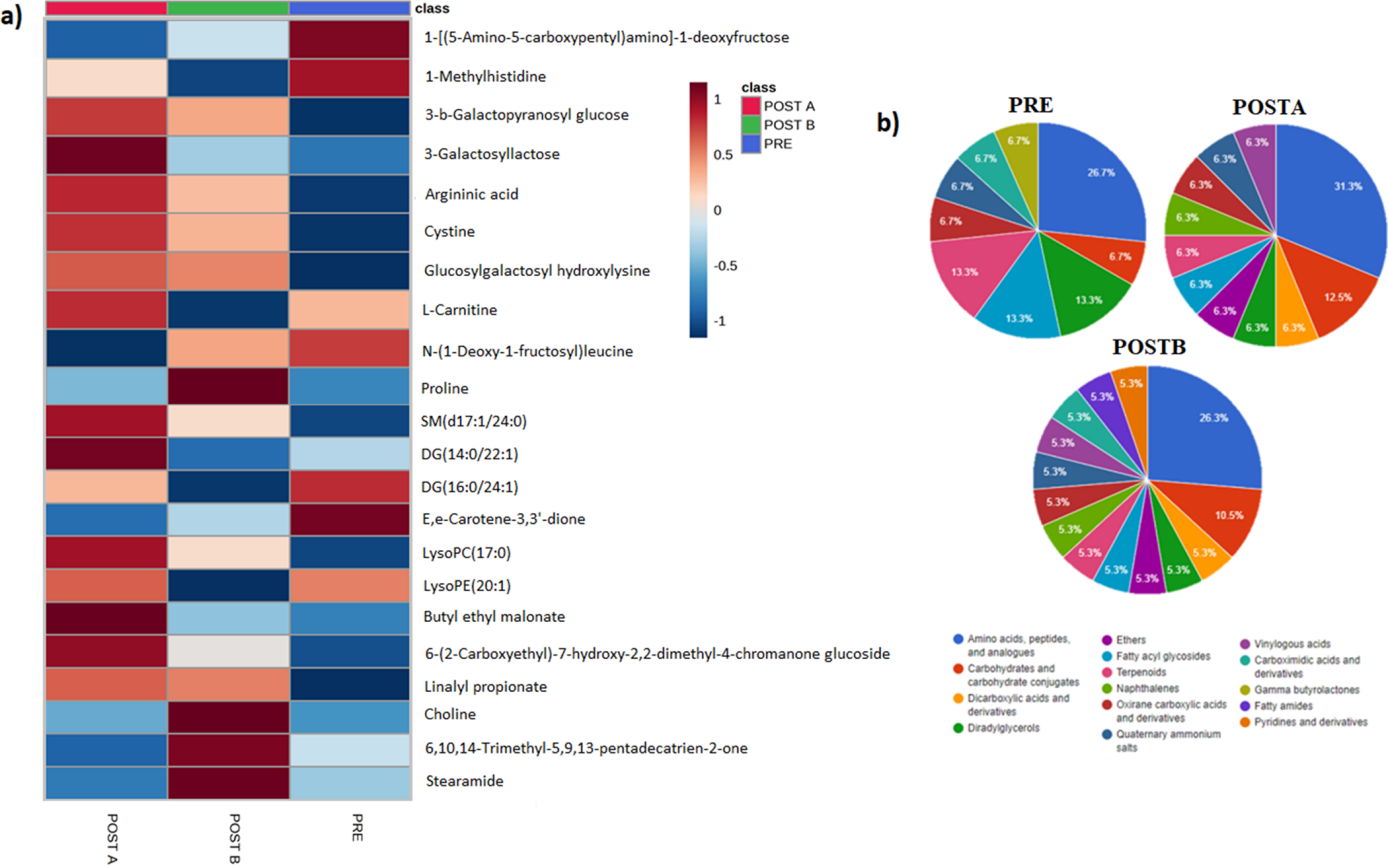
(a) Average abundance heatmap of altered metabolites; (b) most altered subclasses of metabolites when comparing PRE, POSTA, and POSTB groups with the control group.

Figure 2a shows the abundance of the most significant altered metabolites in PRE, POSTA, and POSTB groups determined by RP and HILIC techniques.

**Figure 3:**
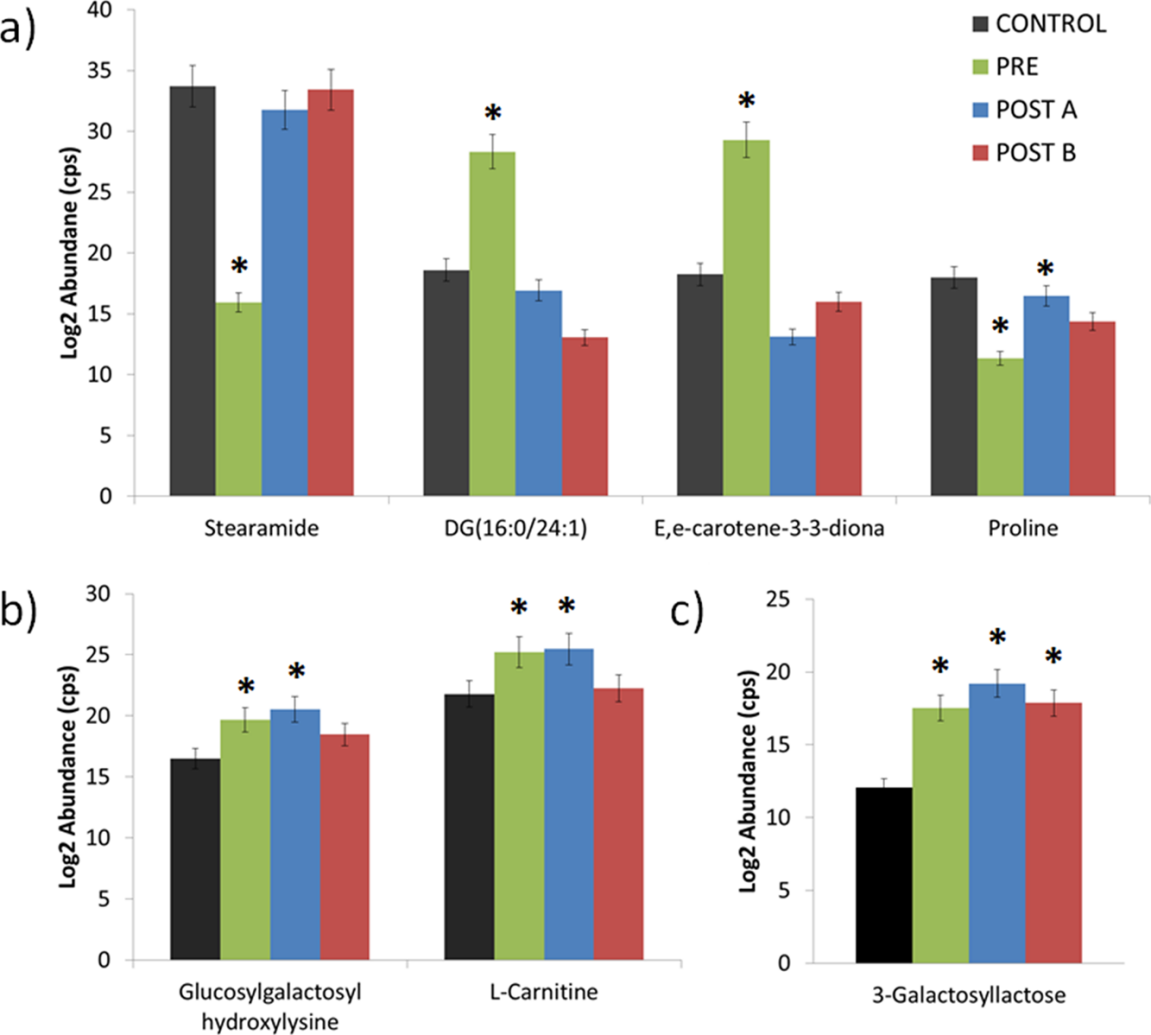
Abundance of altered metabolites after compassion with the control group: (a) metabolites altered in PRE group and nonaltered after surgery; (b) metabolites altered in PRE and POSTA groups and nonaltered in POSTB; (c) metabolites altered in PRE, POSTA, and POSTB groups with similar trends. *Significant differences (ANOVA-Tukey test) respect to control values. Error bars: standard deviation of the mean (SEM).

The abundance of metabolites LysoPC (17:0), stereamide, 6,10,14-trimethyl-5,9,13-pentadecatrien-2-one, and proline (Figure 2a) decreased in LC patients, while their abundance subsequently increased (POSTB) to control levels. Similarly, the levels of DG(14:0/22:1), SM(d17:1/24:0), and l-carnitine (Figure 3) increased in LC patients while they decreased in POSTB with similar levels to those of the control group. In addition, we observed a gradual recovery in the abundance of SM(d17:1/24:0) and stereamide from POSTA to POSTB groups to control levels. 6,10,14-trimethyl-5,9,13-pentadecatrien-2-one, 3-*b*-galactopyranosyl glucose, 3-galactosyllactose, argininic acid, cystine, and proline levels were altered before and immediately after surgery. Although the abundance of these metabolites changed during follow-up in the POSTB group, they were not restored to the control levels. Finally, 1-methylhistidine was a metabolite that continued with the progression of LC after surgery.

The most altered classes of metabolites found in the groups of the study were carboxylic acids (33.3%), organooxygen compounds (23.8%), prenol lipids (14.3%), glycerolipids (9.5%), glycerophospholipids (9.5%), fatty acyls (4.8%), and sphingolipids (4.8%) (Figure S8). Concretely, the most altered subclasses of metabolites in PRE, POSTA, and POSTB compared to the CONTROL group are shown in Figure 2b. Amino acids, peptides, and analogues were the most altered metabolites in the PRE, POSTA, and POSTB groups (26.7, 31.3, and 26.3% of the total altered metabolites, respectively).

Alterations in diacylglycerides (DGs), terpenoids, and fatty acyl glycosides pre- and postsurgery (Figure 2b), suggested perturbations in lipid metabolism, while alterations in carbohydrates and carbohydrate conjugates suggest postoperative changes in carbohydrate metabolism. Similarly, postoperative alterations in dicarboxylic acids and derivatives also suggest that carbohydrate metabolism was affected by the surgical procedure.

Table S5 includes the AUC values of the significantly altered metabolites. In this sense, stearamide (AUC = 0.81) and 3-galactosyllactose (AUC = 0.81) showed AUC values higher than 0.75 when comparing preoperative versus postoperative values. Moreover, galactosyllactose showed a higher value of AUC (AUC = 0.99) when comparing values immediately postsurgery with controls and also at 6 months (AUC = 0.93). When comparing POSTA and control, butyl ethyl malonate (AUC = 0.90), 1-methylhistidine (AUC = 0.89), argininic acid (AUC = 0.77), cystine (AUC = 0.99) and *N*-(1-deoxy-1-fructosyl)leucine (AUC = 0.75) also showed AUC values higher than 0.75. Similarly, in the comparison between POSTB and control, butyl ethyl malonate (AUC = 0.76), 1-methylhistidine (AUC = 0.82), and cystine (AUC = 0.94) presented abundances with AUC values higher than 0.75.

Finally, butyl ethyl malonate (AUC = 0.75), galactosyllactose (AUC = 0.83), 3-*b*-galactopyranosyl glucose (AUC = 0.80), and *N*-(1-deoxy-1-fructosyl)leucine (AUC = 0.76) presented values higher than 0.75 when compared to the PRE and POSTA groups. The Youden index for each ROC analysis is included in the Supporting Information (Table S9).

We can therefore identify a group of metabolites with potential value in the follow-up of LC patients who are surgical candidates. Figure 3 shows the abundance bar graphs of differential metabolites in the study groups. Steramide, DG (16:0/24:1), E,e-carotene-3-3-diona, and proline were perturbed in the PRE group and nonaltered after surgery (Figure 3a); glucosylgalactosyl hydroxyllysine and l-carnitine (Figure 3b) were found altered in PRE and POSTA groups but nonaltered in POSTB; and 3-galactosyllactose (Figure 3c) were altered in PRE, POSTA, and POSTB groups with similar trends.

## Discussion

In this study, we have evaluated metabolic alterations and annotated metabolites that could have potential value as biomarkers for patients with LC undergoing surgical resection.

Metabolomic studies of human serum samples from preoperative and postoperative LC patients are limited in the literature.^8−10^ To our knowledge, this is the first untargeted metabolomic study of serum samples from pre- and postoperative LC patients at two separate time points after surgery (1 month, POSTA and 3–6 months, POSTB). Moreover, the combination of HILIC and RP chromatography provides a new approach since most of the published works reported the use of reversed-phase ultra-high liquid chromatography (RP-UHPLC-MS) or gas chromatography (GC-MS).^8−10^

### Metabolite Alterations in PRE, POSTA, and POSTB Compared with the CONTROL Group

We found that the majority of altered metabolites against the control increased in the pre- and postoperative groups at 1 month (POSTA) and 3–6 months after surgery (POSTB) when compared to a control group, including amino acids, fatty acyls, carbohydrates, and lipids. Yang et al. reported similar results for amino acids, fatty acids, and other specific lipids in preoperative and postoperative LC patient samples.^8^ Proline levels decreased before and immediately after surgery but returned to baseline during the follow-up. Several authors have found diminished levels of amino acids in preoperative LC patient samples^10^ although other authors have described augmented levels of these metabolites. Alterations in amino acids are related to proliferation and survival of cancer cells under genotoxic, oxidative, and nutritional stress.^13^

We found increased levels of the carbohydrate 3-galactosyllactose in consonance with data reported by Ahmed et al. They found increased levels of several carbohydrates in the serum of preoperative LC patients.^10^ It is well known that tumor cells have an impact on carbohydrate metabolism, which in turn is linked to unregulated cellular proliferation, rapid proliferation, and metastasis.^14^ Alterations in several lipids, such as fatty acids, lysophosphatidylcholines, lysophosphatidylethanolamines, phosphatidylcholines, sphingomyelins, and glycerides have also been described in preoperative samples of LC patients.^8−10^ In our study, DGs, fatty acyls, sphingomyelins, and prenol lipids were altered in preoperative samples. Specifically, the abundances of DG(14:0/22:1) and DG(16:0/24:1) were higher in this group. DGs contribute to energy storage, energy metabolism, and signal transduction and are components of cellular membranes, which act as building blocks for glycerophospholipids and as lipid second messengers. Some authors have observed diminished levels of tryacylglycerides (TGs) in preoperative LC patients.^8^ Although we did not find altered levels of TGs in our study, the degradation of these metabolites could explain the increase in DGs in preoperative LC serum samples. On the other hand, prenol lipid levels such as linalyl propionate and E,e-carotene-3,3′dione were higher in PRE patients. Prenol lipids are important for health due to their antioxidant effect. In this sense, Yang et al., also found alterations in prenol lipids in preoperative LC patients.^8^ Lipids are the main components of biological membranes and signaling molecules needed for proliferation, survival, invasion, metastasis, and interaction with the tumor microenvironment.^15^ Concentrations of other lipids, such as glucosylgalactosyl hydroxyllysine, also increased preoperatively. We also found increased levels of l-carnitine in the same group. l-carnitine is involved in numerous metabolic pathways, including the β-oxidation of fatty acids, where FAs are broken into acetyl-CoA, which then enters the TCA to aid ATP generation. Accumulated evidence suggests that many cancer cells reprogram FAO and rely on this process for proliferation, survival, drug resistance, or metastasis.^16^ In several works, authors have found increased levels of l-carnitine in serum from preoperative LC patients.^9,10^

### Metabolites Altered in PRE Group and Nonaltered after Surgery

The metabolites stearamide, DG (16:0/24:1), E,e-carotene-3-3-diona, and proline were found altered before surgery in PRE patients but returned to baseline in both postoperative groups. This finding could indicate that possible curative resection could influence the levels of these metabolites in LC patients. Moreover, the analysis of ROC curves showed a good value of AUC for stearamide (AUC = 0.81). Several previous studies considered stearamide as a LC-related metabolite (http://cosbi4.ee.ncku.edu.tw/LCMD/). This metabolite could continue to be studied in future studies to verify its role as a possible biomarker of successful resection.

### Metabolites Altered in PRE and POSTA Groups and Nonaltered in POSTB

We found several altered metabolites before and immediately after surgery but returned to baseline after 3–6 months of surgery. The abundance of glucosylgalactosy hydroxyllysine, l-carnitine, and proline was significantly different in PRE and POSTA against the control group, but these changes were not significant in the POSTB group. In addition, the metabolites glucosylgalactosyl hydroxyllysine and l-carnitine showed good specificity and sensitivity with AUC values of 0.89 and 0.79, respectively, in POSTA groups (Table S5).

### Metabolites Altered in PRE and POSTB Groups with Similar Trends, but Comparable with the Control Group in POSTA

We found that choline and SM (d17:1/24:0) were altered in the POSTB group when compared with the control group, but they were similar to the control in POSTA. However, no good values of AUC were found in the ROC analysis. In spite of that, the dysregulation of glycerophospholipids and SM in LC has been extensively reported.^17^

### Metabolites Altered in PRE, POSTA, and POSTB Groups with Similar Trends

DG (14:0/22:1) and 3-galactosyllactose were significantly increased in PRE, and they remained augmented in POSTA and POSTB groups. Alterations in these metabolites persisted after surgery. In addition, 3-galactosyllactose presented an AUC value higher than 0.75 in the PRE, POSTA, and POSTB groups (Table S6). Hypothetically, these metabolites could indicate the failure of the surgery. Regarding 3-galactosyllactose, the rapid proliferation of cancer cells increases their nutritional requirements, resulting in the high expression of lectin-like receptors on the surface of cancer cells that have a strong affinity for mannosyl and galactosyl groups.^18^

## Study Limitations

It is important to consider some limitations of this work. First, the number of samples, especially the number of postoperative patients, is relatively small. In addition, samples from the same patients that were collected 3 and 6 months after the intervention were included in order to consider a greater number of samples in the POSTB group. NSCLC patients had different NSCL subtypes, and this variability could affect the results. In this sense, to secure the conclusions, a validation study in a larger population would be necessary to ascertain these new insights. On the other hand, there is a lack of studies analyzing the impact of surgery and inflammation on the metabolome of healthy people. This fact could act as a confounding factor that may be difficult to control in further surgery-based analysis.

## Conclusions

In this work, we have applied a combined metabolomic platform based on RP and HILIC chromatography coupled with QTOF to determine altered metabolites in serum samples from preoperative and postoperative LC patients, one month and 3–6 months after surgery. We found alterations in the levels of steramide, DG (16:0/24:1), E,e-carotene-3–3-diona, proline glucosylgalactosyl hydroxyllysine, and l-carnitine in serum samples from LC patients, which returned to those of a baseline control group 3–6 months after surgery. Furthermore, 3-galactosyllactose levels remained altered after the intervention in some patients. A deeper study of these metabolites as biomarkers could provide new insights about the possible recovery or progression of LC after surgery with curative intent. To the best of our knowledge, this work is the first to address the metabolic changes in serum samples from preoperative and postoperative patients collected at two different time points using the combination of RP and HILIC chromatography in order to provide a wide metabolic net, including metabolites of different polarities.

## Data Availability

The data sets analyzed during the current study are available at ftp://massive.ucsd.edu/MSV000092213/ (MassIVE ID MSV000092213).

## Supporting Information Available

Clinical characteristics of patients included in the study; batch recursive feature extraction parameters; coefficient of variation (CV) of metabolites calculated in quality control (QC) samples; PLS-DA Q2 and R2 values of pairwise comparison of CONTROL, PRE, POSTA, and POSTB groups; altered metabolites ordered by class; MS/MS fragments of altered metabolites for accurate identification; coefficient of variation of the abundance of altered metabolites per group; Venn diagram results; sensitivity and specificity farthest to diagonal line (Youden index) for ROC curves; PCA plots showing the clustering of the QCs samples; metabolomic profiles of extract samples; 2D-PLSDA plot pairwise PRE-CONTROL comparison; 2D-PLSDA plot pairwise POSTA-CONTROL comparison; 2D-PLSDA plot pairwise POSTB-CONTROL comparison; 2D-PLSDA plot pairwise PRE-POSTA comparison; 2D-PLSDA plot pairwise PRE-POSTA comparison; and main altered classes of metabolites in PRE, POSTA and POST B groups compared to CONTROL (PDF)

## Dedication

The study was performed in accordance with the principles contained in the Declaration of Helsinki and approved by the Ethical Committee of the Andalusian Government (Ethical code num. 1898-N-21). The data of the patients are anonymized in a database with hierarchical access control in order to guarantee secure information access.

## Abbreviations

DGs: diacylglycerides
ESI: electrospray ionization
GC: gas chromatography
HPLC: high-performance liquid chromatography
LC: lung cancer
LysoPC: lysophosphocholine
*m*/*z*: mass/charge
MS: mass spectrometry
NSCLC: non-small cell lung cancer
PC: phosphocholine
PCA: principal component analysis
PLS-DA: partial least square discriminant analysis
Q: quadrupole
QQQ: triple quadrupole
ROC: receiver operator characteristic
SCLC: small cell lung cancer
SM: sphingomyelin
TAGs: triacylglycerides
TOF: time of flight
