# Supplementary material for: Untargeted Metabolomic Study of Lung Cancer Patients after Surgery with Curative Intent
Source: J Proteome Res. 2023 Oct 16;22(11):3499–507. doi: 10.1021/acs.jproteome.3c00356 (PMC10629266; doi:10.1021/acs.jproteome.3c00356)

Supporting Information

Untargeted metabolomic study of lung cancer patients after surgery with curative intent

Saida Sanchez-Espirilla[1,2,f], Antonio Pereira-Vega[3f], Belén Callejón-Leblic[1*], Isabel Díaz-Olivares, Rafael Santana[4], Carolina Gotera Rivera[4], José Luis Gómez-Ariza[1], José Luis López-Campos[5,6], Ana Isabel Blanco-Orozco[5], Luis Seijo[7], María Rodríguez[7], Luis Alejandro Padrón Fraysse[3], Ángeles Herrera-Chilla[3], Germán Peces-Barba[4] and Tamara García Barrera[1*].

[1]Department of Chemistry, Research Center for Natural Resources, Health and the Environment (RENSMA). Faculty of Experimental Sciences, University of Huelva, Campus El Carmen, Fuerzas Armadas Ave., 21007, Huelva, Spain. [2]Department of Chemistry, Faculty of Sciences, National University of San Antonio Abad of Cusco, Av. de La Cultura, 773, Cusco, Peru. [3]Pneumology Area of the Juan Ramón Jiménez Hospital, Ronda Norte, s/n, 21005, Huelva, Spain. [4]IIS Jiménez Díaz Foundation. ISCIII-CIBERES. Reyes Católicos Ave., 28040, Madrid, Spain. [5]Medical-Surgical Unit of Respiratory Diseases. Institute of Biomedicine of Seville (IBiS), Antonio Maura Montaner, 41013, Seville. Virgen del Rocío University Hospital/University of Seville, Manuel Siurot, s/n, 41013. Sevilla, Spain. [6]Center for Biomedical Research in Respiratory Diseases Network (CIBERES). Monforte de Lemos Ave., 28029, Carlos III Health Institute, Madrid, Spain. [7]University Clinic of Navarra, Monforte de Lemos Ave., 28029, Madrid, Spain.

***Corresponding authors: belen.callejon@dqcm.uhu.es; tamara@dqcm.uhu.es**

[f]These authors contributed equally and should be considered co-first authors.

Supporting Contents	
Table S1	Clinical Characteristics of patients included in the study. PRE: samples from preoperative lung cancer patients; POSTA: samples from postoperative lung cancer patients collected at 1 month of surgery; POSTB: samples from postoperative lung cancer patients collected at 3-6 months of surgery
Table S2	Batch Recursive Feature Extraction parameters (UHPLC-QTOF-MS analysis)
Table S3	Coefficient of variation (CV) of metabolites calculated in quality control (QC) samples.
Table S4	PLS-DA Q^2 and R^2 values of pairwise comparison of CONTROL, PRE, POSTA and POSTB groups.
Table S5	Altered metabolites ordered by class
Table S6	MS/MS fragments of altered metabolites for accurate identification.
Table S7	Coefficient of variation of the abundance of altered metabolites per group.
Table S8	Venn diagram results. Common metabolites in the study groups.
Table S9	Sensitivity and Specificity farthest to diagonal line (Youden index) for ROC curves.
Figure S1	PCA plots showing the clustering of the QCs samples
Figure S2	Metabolomic profiles of extract samples
Figure S3	2D-PLSDA plot pairwise PRE-CONTROL comparison
Figure S4	2D-PLSDA plot pairwise POSTA-CONTROL comparison
Figure S5	2D-PLSDA plot pairwise POSTB-CONTROL comparison
Figure S6	2D-PLSDA plot pairwise PRE-POSTA comparison
Figure S7	2D-PLSDA plot pairwise PRE-POSTA comparison
Figure S8	Main altered classes of metabolites in PRE, POSTA and POST B groups compared to CONTROL

Table S1. Clinical Characteristics of patients included in the study. PRE: samples from preoperative lung cancer patients; POSTA: samples from postoperative lung cancer patients collected at 1 month of surgery; POSTB: samples from postoperative lung cancer patients collected at 3-6 months of surgery; DM= Diabetes Mellitus; AH: Arterial Hypertension; DLP: Dyslipidemia; COPD: Chronic Obstructive Pulmonary Disease.

**Clinical characteristics**		**CONTROL (n= 35)**	**PRE (n=48)**	**POSTA (n=15)**	**POSTB (n=17)**
Gender	Male	21	29	8	12
	Female	14	19	7	6
Age (years ± SD)		56.3 ± 13.1	65.7 ± 10.7	62.3 ± 9.1	67.33 ± 8.1
Smoking habits	Non-smoker	16	11	0	0
	Smoker	0	13	8	9
	Exsmoker	19	24	7	8
NSCLC Type	Adenocarcinoma	-	32	15	17
	Lepidic adenocarcinoma	-	1	-	-
	Adenosquamous	-	1	-	-
	Carcinoid	-	2	-	-
	Carcinoma	-	6	-	-
	Squamous cell carcinoma	-	1	-	-
	Epidermoid	-	4	-	-
	Typical Carcinoid Tumor	-	1	-	-
Comorbidities	DM	8	5	0	1
	AH	0	20	5	8
	DLP	0	17	2	4
	COPD	0	15	8	5
Surgery	Lobectomy	-	32	14	15
	Segmentectomy	-	15	0	1
	Wedge resertion	-	2	1	1

Table S2. Batch Recursive Feature Extraction parameters (UHPLC-QTOF-MS analysis)

<table>
<tr><th colspan="2">Batch Recursive Feature Extraction</th></tr>
<tr><th colspan="2">Extraction</th></tr>
<tr><td>Peak weight</td><td>> 600 counts</td></tr>
<tr><th colspan="2">Ion species</th></tr>
<tr><td>Positive Mode</td><td>$[M+H]^+$, $[M+Na]^+$</td></tr>
<tr><td>Negative Mode</td><td>$[M-H]^-$, $[M-Cl]^-$</td></tr>
<tr><td>Integration</td><td>Agile 2</td></tr>
<tr><th colspan="2">Alignment Parameters</th></tr>
<tr><td>RT Tolerance</td><td>0.00% ± 0.3 min</td></tr>
<tr><td>Mass Tolerance</td><td>20 ppm ± 2.00 mDa</td></tr>
<tr><th colspan="2">Molecular Feature Extraction Filters</th></tr>
<tr><td>Score (MFE)</td><td>> 97</td></tr>
<tr><th colspan="2">Tolerance and EIC</th></tr>
<tr><td>Masses</td><td>± 10.00 ppm</td></tr>
<tr><td>RT</td><td>± 0.300 min</td></tr>
<tr><td>Possible m/z</td><td>Symmetric (ppm)</td></tr>
<tr><th colspan="2">Peak Filter</th></tr>
<tr><td>Absolute area</td><td>> 20000 counts</td></tr>
<tr><td>Chromatogram Format</td><td>Centroid</td></tr>
<tr><th colspan="2">Peak Spectrum</th></tr>
<tr><td>Average scans</td><td>10 % of peak height</td></tr>
<tr><th colspan="2">Find by ion filters</th></tr>
<tr><td>Score (Tgt)</td><td>> 97</td></tr>
<tr><th colspan="2">Entities</th></tr>
<tr><td colspan="2">IR-UHPLC-QTOF-MS</td></tr>
<tr><td rowspan="2">Number of entities found</td><td>Positive Mode: 333</td></tr>
<tr><td>Negative Mode: 1218</td></tr>
<tr><td rowspan="2">Number of significant entities found</td><td>Positive Mode: 333</td></tr>
<tr><td>Negative Mode: 155</td></tr>
<tr><td colspan="2">HILIC-UHPLC-QTOF-MS</td></tr>
<tr><td>Number of entities found</td><td>Positive Mode: 683</td></tr>
<tr><td>Number of significant entities found</td><td>Positive Mode: 54</td></tr>
</table>

Table S3. Coefficient of variation (CV) of metabolites calculated in quality control (QC) samples. Lysophosphatidylcholine (LPC), lysophosphatidylethanolamine (LPE),Sphingomyeline (SM); dyacylglyceride (DG)

**Compound**	**%CV in QC (n=8)**
6-(2-Carboxyethyl)-7-hydroxy-2,2-dimethyl-4-chromanone glucoside	0.64
6,10,14-Trimethyl-5,9,13-pentadecatrien-2-one	4.54
Butyl ethyl malonate	3.97
Choline	3.94
DG(14:0/22:1)	4.11
DG(16:0/24:1)	3.34
E,e-Carotene-3,3'-dione	6.19
Linalyl propionate	5.38
LPC(17:0)	0.62
LPE(20:1)	2.20
SM(d17:1/24:0)	1.08
Stearamide	1.15
1-[(5-Amino-5-carboxypentyl)amino]-1-deoxyfructose	2.85
1-Methylhistidine	9.92
3-b-Galactopyranosyl glucose	1.71
3-Galactosyllactose	0.62
Cystine	0.74
Glucosylgalactosyl hydroxylysine	2.21
L-Carnitine	3.21
N-(1-Deoxy-1-fructosyl)leucine	1.25
Proline	1.08

Table S4. PLS-DA Q^2 and R^2 values of pairwise comparison of CONTROL, PRE, POSTA and POSTB groups.

Parameters Q^2/R^2Y						
Analytical Methodology	Extract	PRE vs CONTROL	POSTA vs CONTROL	POSTB vs CONTROL	PRE vs POSTA	PRE vs POSTB
ESI(+)-RP-UHPLC-QTOF-MS	MeOH:H_2O (8:1 v/v)	0.85/0.99	0.76/0.99	0.64/0.99	0.39/0.93	0.58/0.99
ESI(+)-RP-UHPLC-QTOF-MS	ACN:MeOH (6:4 v/v)	0.73/0.92	0.77/0.99	0.78/0.99	0.20/0.82	0.65/0.98
ESI(-)-RP-UHPLC-QTOF-MS	MeOH:H_2O (8:1 v/v)	0.84/0.99	0.45/0.97	0.65/0.99	0.47/0.89	0.28/0.91
ESI(+)-HILIC-UHPLC-QTOF-MS	MeOH:H_2O (4:1 v/v)	0.83/0.93	0.95/0.98	0.89/0.96	0.31/0.87	0.11/0.82

Table S5. Altered metabolites ordered by class. RT: retention time, FC: Fold change, N.S.: Not significant, lysophosphatidylcholine (LPC), lysophosphatidylethanolamine (LPE), Sphingomyeline (SM); dyacylglyceride (DG); Experimental Mass (EM); Theorethical Mass (TM); Methodology (MD); Ionization Mode (IM); *p*-value obtained from one way ANOVA followed by Tuckey Test and corrected by Benjamini-Hochberg multiple post-correction.

Metabolites	PRE vs CONTROL			POSTA vs CONTROL			POSTB vs CONTROL			PRE vs POSTA			PRE vs POSTB			EM	TM	MD	IM	Class
	FC	P	AUC	FC	P	AUC	FC	P	AUC	FC	P	AUC	FC	P	AUC					
**1-[(5-Amino-5-carboxypentyl)amino]-1-deoxyfructose**	1.05	NS	-	0.94	NS	-	0.98	NS	-	1.12	0.00	0.75	1.07	NS	-	308.1589	308.1584	HILIC	ESI +	Organooxygen compounds
**1-Methylhistidine**	0.88	NS	-	0.86	0.00	0.79	0.81	0.00	0.82	1.03	NS	-	1.08	0.02	0.70	169.0854	169.0851	HILIC	ESI +	Carboxylic acids and derivatives
**Cystine**	1.06	NS	-	1.21	0.00	1.00	1.29	0.00	0.94	0.96	NS	-	0.97	NS	-	240.024	240.0238	HILIC	ESI +	Carboxylic acids and derivatives
**N-(1-Deoxy-1-fructosyl)leucine**	1.13	NS	-	0.91	0.01	0.75	1.10	NS	-	1.23	0.00	0.76	1.03	NS	-	293.1479	293.1475	HILIC	ESI +	Carboxylic acids and derivatives
**Butyl ethyl malonate**	1.35	NS	-	4.34	0.02	0.90	1.66	0.02	0.76	0.31	0.04	0.75	0.81	0.02	0.55	188.105	188.1049	RP	ESI-	Carboxylic acids and derivatives
**Argininic acid**	1.01	NS	-	1.15	0.01	0.77	1.14	0.03	0.69	0.96	NS	-	0.97	NS	-	175.0959	175.0957	HILIC	ESI +	Carboxylic acids and derivatives
**Proline**	0.63	0.00	0.70	0.63	0.02	0.72	0.80	NS	-	1.00	NS	-	0.79	NS	-	115.0639	115.0633	HILIC	ESI +	Carboxylic acids and derivatives
**Stearamide**	0.38	0.02	0.81	0.72	NS	-	0.99	NS	-	1.01	NS	-	1.14	NS	-	283.2908	283.2875	RP	ESI+	Carboximidic acids and derivatives
**3-b-Galactopyranosyl glucose**	1.16	0.02	0.66	1.26	NS	-	1.22	0.00	0.89	0.85	0.00	0.80	0.87	0.01	0.72	342.1189	342.1162	HILIC	ESI +	Epoxides
**Glucosylgalactosyl hydroxylysine**	1.19	0.00	0.72	1.24	0.00	0.87	1.12	NS	-	0.95	0.04	0.69	1.07	NS	-	486.206	486.2061	HILIC	ESI +	Fatty Acyls
**DG(14:0/22:1)**	2.06	0.02	0.67	6.13	0.00	0.60	1.29	0.04	0.55	0.34	0.03	0.74	1.60	NS	-	622.5529	622.5536	RP	ESI+	Glycerolipids
**DG(16:0/24:1)**	1.68	0.03	0.65	0.85	NS	-	0.43	NS	-	1.98	NS	-	3.91	NS	-	678.6113	678.6162	RP	ESI+	Glycerolipids
**LysoPC(17:0)**	0.62	NS	-	0.90	NS	-	1.00	NS	-	0.69	0.01	0.56	0.62	0.04	0.5	509.35	509.3481	RP	ESI+	Glycerophospholipids
**LysoPE(20:1)**	1.70	NS	-	1.50	NS	-	0.21	NS	-	1.13	NS	-	7.98	0.02	0.71	507.3325	507.3325	RP	ESI+	Glycerophospholipids
**L-Carnitine**	1.16	0.00	0.70	1.18	0.00	0.79	1.02	NS	-	0.99	NS	-	1.04	NS	-	161.1056	161.1052	HILIC	ESI +	Organonitrogen compounds
**Choline**	0.87	NS	-	1.15	NS	-	1.97	0.02	0.68	0.76	NS	-	0.44	0.01	0.61	103.1003	103.1	RP	ESI+	Organonitrogen compounds
**3-Galactosyllactose**	1.45	0.00	0.89	1.59	0.00	0.99	1.48	0.00	0.93	0.91	0.00	0.83	0.98	NS	-	504.1683	504.1690	HILIC	ESI +	Organooxygen compounds
**6-(2-Carboxyethyl)-7-hydroxy-2,2-dimethyl-4-chromanone glucoside**	1.06	NS	-	5.36	0.00	0.65	2.29	0.00	0.69	0.20	0.01	0.53	0.46	0.01	0.54	426.1522	426.1526	RP	ESI-	Organooxygen compounds
**6,10,14-Trimethyl-5,9,13-pentadecatrien-2-one**	0.63	0.00	0.55	0.12	NS	-	0.29	NS	-	1.03	NS	-	0.43	NS	-	262.2275	262.2297	RP	ESI+	Prenol lipids
**E,e-Carotene-3,3'-dione**	1.79	0.03	0.62	0.45	NS	-	0.78	NS	-	3.97	NS	-	2.29	NS	-	564.398	564.3967	RP	ESI+	Prenol lipids
**Linalyl propionate**	2.10	NS	-	1.03	NS	-	1.14	0.00	0.60	1.01	NS	-	1.11	0.05	0.54	210.1618	210.162	RP	ESI-	Prenol lipids
**SM(d17:1/24:0)**	1.76	NS	-	1.14	NS	-	0.73	0.01	0.63	1.55	NS	-	2.41	0.00	0.62	800.6704	800.6771	RP	ESI+	Sphingolipids

Table S6. MS/MS fragments of altered metabolites for accurate identification.

Metabolites	MS/MS fragments (m/z) CE (10-40V)
**1-[(5-Amino-5-carboxypentyl)amino]-1-deoxyfructose**	309.1656: 205.1179;130.0858; 84,0812
**1-Methylhistidine**	170.0932; 124.0869; 107,0606; 97.0761
**3-b-Galactopyranosyl glucose**	325.1125; 181.0705; 163.0598; 59.0126
**3-Galactosyllactose**	505.1765; 343.1236; 325.1125;145.0491
**6-(2-Carboxyethyl)-7-hydroxy-2,2-dimethyl-4-chromanone glucoside**	425.1440; 407.1337; 263.0915; 245.0811; 161.0443
**6,10,14-Trimethyl-5,9,13-pentadecatrien-2-one**	263.2369; 245.2273; 175.1487; 107.0863; 69.0702
**Argininic acid**	158.0921; 117.0542; 99.0440; 71.0487; 60.0551
**Butyl ethyl malonate**	187.0966; 131.0342; 113.0233;87.0443; 45.0341
**Choline**	104.1068; 60.0819; 58.0654
**Cystine**	241.0308; 151.9837; 122.0282; 120.0118
**DG(14:0/22:1)**	640.5872; 605.5504; 397.3676; 283.2265
**DG(16:0/24:1)**	696.6504; 661.6132; 423.3841; 313.2747
**E,e-Carotene-3,3'-dione**	565.4038; 537.4089; 411.2679; 345.2213; 149.0958
**Glucosylgalactosyl hydroxylysine**	469.2031; 423.1975; 307.1502; 163.0602; 146.0815
**L-Carnitine**	162.1119; 103.0384; 85.0281; 60.0811
**Linalyl propionate**	137.1335; 135.1183; 73.0291; 51.0237
**LysoPC(17:0)**	532.3373; 473.2642; 349.2710; 327.2890; 104.1069
**LysoPE(20:1)**	508.3395; 465.2971; 293.2843; 198.0521; 75.0441;44.0493
**N-(1-Deoxy-1-fructosyl)leucine**	294.1551; 248.1495; 230.1389; 144.1021; 98.0966; 57.0704
**Proline**	116.0751; 70.0671
**SM(d17:1/24:0)**	823.6661; 764.5929; 640.6005
**Stearamide**	284.2949; 267.2682; 266.2841; 249.2578; 239.3743

Table S7. Coefficient of variation of the abundance of altered metabolites per group.

Coefficient of variation (CV%)				
**Metabolites**	**CONTROL**	**PRE**	**POSTA**	**POSTB**
**1-[(5-Amino-5-carboxypentyl)amino]-1-deoxyfructose**	14.3	10.0	2.9	8.3
**1-Methylhistidine**	7.2	7.9	7.1	8.3
**3-b-Galactopyranosyl glucose**	8.2	9.5	4.8	7.0
**3-Galactosyllactose**	4.6	4.3	2.5	4.0
**Argininic acid**	5.5	4.7	3.3	4.6
**Cystine**	4.7	5.7	0.9	2.9
**Glucosylgalactosyl hydroxylysine**	4.7	4.9	3.9	3.2
**L-Carnitine**	4.3	4.4	3.9	4.8
**N-(1-Deoxy-1-fructosyl)leucine**	9.6	8.8	3.7	8.6
**Proline**	9.0	11.4	9.1	11.5
**DG(14:0/22:1)**	2.7	2.9	2.9	2.2
**DG(16:0/24:1)**	5.1	2.9	4.7	3.9
**E,e-Carotene-3,3'-dione**	5.3	15.2	4.1	5.5
**LysoPC(17:0)**	5.5	4.3	5.0	4.6
**LysoPE(20:1)**	5.3	14.9	3.7	5.0
**SM(d17:1/24:0)**	5.5	5.0	3.5	5.6
**Butyl ethyl malonate**	2.1	2.9	1.4	2.6
**6-(2-Carboxyethyl)-7-hydroxy-2,2-dimethyl-4-chromanone glucoside**	3.9	14.5	4.8	2.7
**Linalyl propionate**	4.9	5.1	5.2	7.2
**Choline**	4.4	3.4	2.8	4.2
**6,10,14-Trimethyl-5,9,13-pentadecatrien-2-one**	13.7	9.3	6.7	6.9
**Stearamide**	5.7	8.6	5.7	7.1

Table S8. Venn diagram results. Common metabolites in the study groups.

Groups	Commom metabolites	Metabolites
POSTA<br>POSTB<br>PRE	2	3-Galactosyllactose<br>DG(14:0/22:1)
POSTA<br>PRE	3	Glucosylgalactosyl hydroxylysine<br>Proline<br>L-Carnitine
POSTB<br>PRE	1	3-b-Galactopyranosyl glucose
POSTA<br>POSTB	5	1-Methylhistidine<br>Butyl ethyl malonate<br>Cystine<br>6-(2-Carboxyethyl)-7-hydroxy-2,2-dimethyl-4-chromanone glucoside<br>Argininic acid

Table S9. Sensitivity and Specificity farthest to diagonal line (Youden index) for ROC curves.

PRE/CONTROL			
**Compounds**	**Cutoff**	**Sensitivity**	**Specificity**
3-Galactosyllactose	17.5	0.792(0.667-0.886)	0.903(0.774-0.968)
Steramide	17.1	0.75(0.635-0.875)	0.818(0.727-0.925)
**POST A/CONTROL**			
**Compounds**	**Cutoff**	**Sensitivity**	**Specificity**
Cystine	23.3	0.923(0.769-1)	1(1-1)
3-Galactosyllactose	18.1	1(1-1)	0.968(0.871-1)
3-b-Galactopyranosyl glucose	21.8	0.923(0.769-1)	0.903(0.79-1)
Glucosylgalactosyl hydroxylysine	18.1	0.769(0.538-0.963)	0.806(0.645-0.903)
L-Carnitine	22.7	0.769(0.538-1)	0.645(0.499-0.759)
1-Methylhistidine	21.8	0.692(0.421-0.923)	0.774(0.66-0.903)
Argininic acid	22.9	0.846(0.692-1)	0.806(0.66-0.903)
Butyl ethyl malonate	19.4	0.75(0.54-0.96)	0.848(0.727-0.97)
**POST B/CONTROL**			
**Compounds**	**Cutoff**	**Sensitivity**	**Specificity**
Cystine	23	0.833(0.667-1)	0.935(0.839-1)
3-Galactosyllactose	17.8	0.889(0.749-1)	0.935(0.854-1)
3-b-Galactopyranosyl glucose	21.7	0.778(0.611-0.974)	0.903(0.774-1)
1-Methylhistidine	21.3	0.667(0.471-0.889)	0.903(0.806-1)
Butyl ethyl malonate	19.5	0.706(0.471-0.854)	0.758(0.62-0.879)
**PRE/POST A**			
**Compounds**	**Cutoff**	**Sensitivity**	**Specificity**
3-Galactosyllactose	18.8	0.729(0.593-0.854)	0.769(0.498-1)
3-b-Galactopyranosyl glucose	22.2	0.688(0.583-0.792)	0.846(0.692-1)
1-[(5-Amino-5-carboxypentyl)amino]-1-deoxyfructose	21.5	0.625(0.51-0.75)	0.923(0.769-1)
N-(1-Deoxy-1-fructosyl)leucine	21.1	0.708(0.583-0.854)	0.692(0.462-0.923)
E,e-Carotene-3,3'-dione	16	0.688(0.572-0.823)	0.833(0.583-1)
Butyl ethyl malonate	19.2	0.792(0.646-0.886)	0.583(0.333-0.833)

Figure S1. PCA plots showing the clustering of the QCs samples in a) aqueous extract determined by ESI(+)-RP-UHPLC-QTOF-MS; b) polar extract determined by ESI(-)-RP-UHPLC-QTOF-MS; c) organic extract determined by ESI(+)-RP-UHPLC-QTOF-MS; and d) polar extract determined by ESI(+)-HILIC-UHPLC-QTOF-MS. Black dots :CONTROL, green dots: PRE, blue dots: POSTA, red dots: POSTB, yellow dots: QC samples.

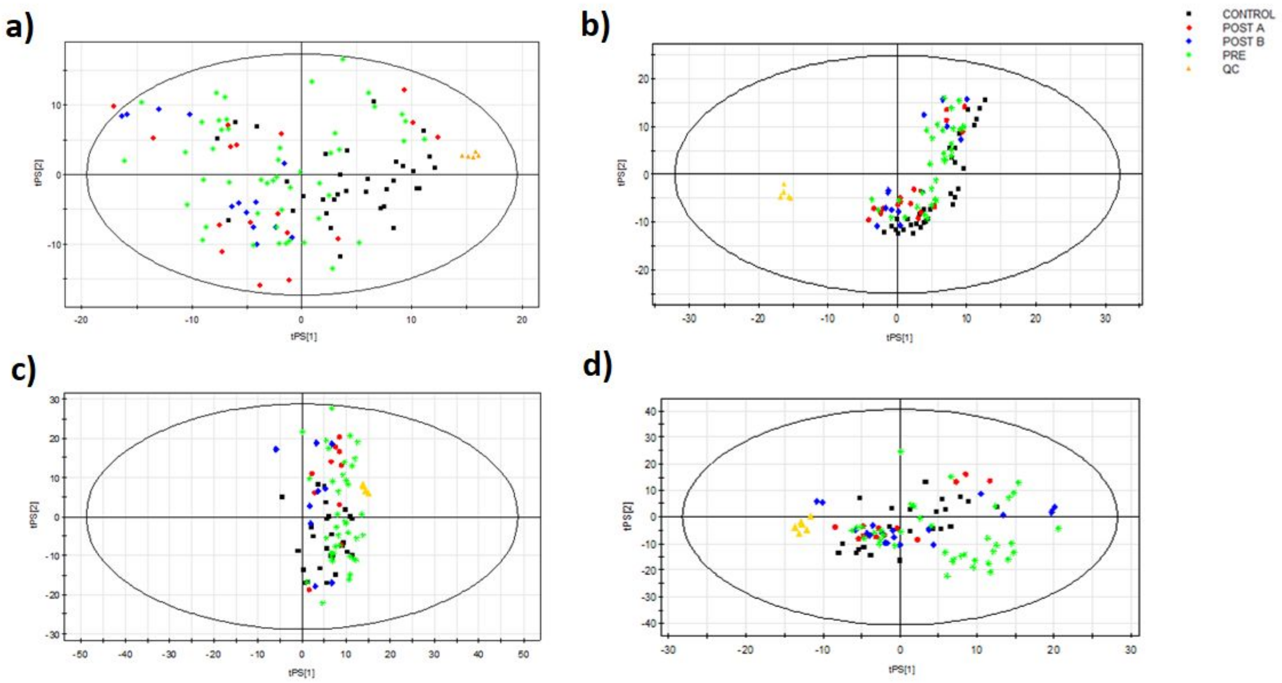

Figure S2. Metabolomic profiles of extract samples a) aqueous extract determined by ESI(+)-RP-UHPLC-QTOF-MS; b) polar extract determined by ESI(-)-RP-UHPLC-QTOF-MS; c) organic extract determined by ESI(+)-RP-UHPLC-QTOF-MS; and d) polar extract determined by ESI(+)-HILIC-UHPLC-QTOF-MS.

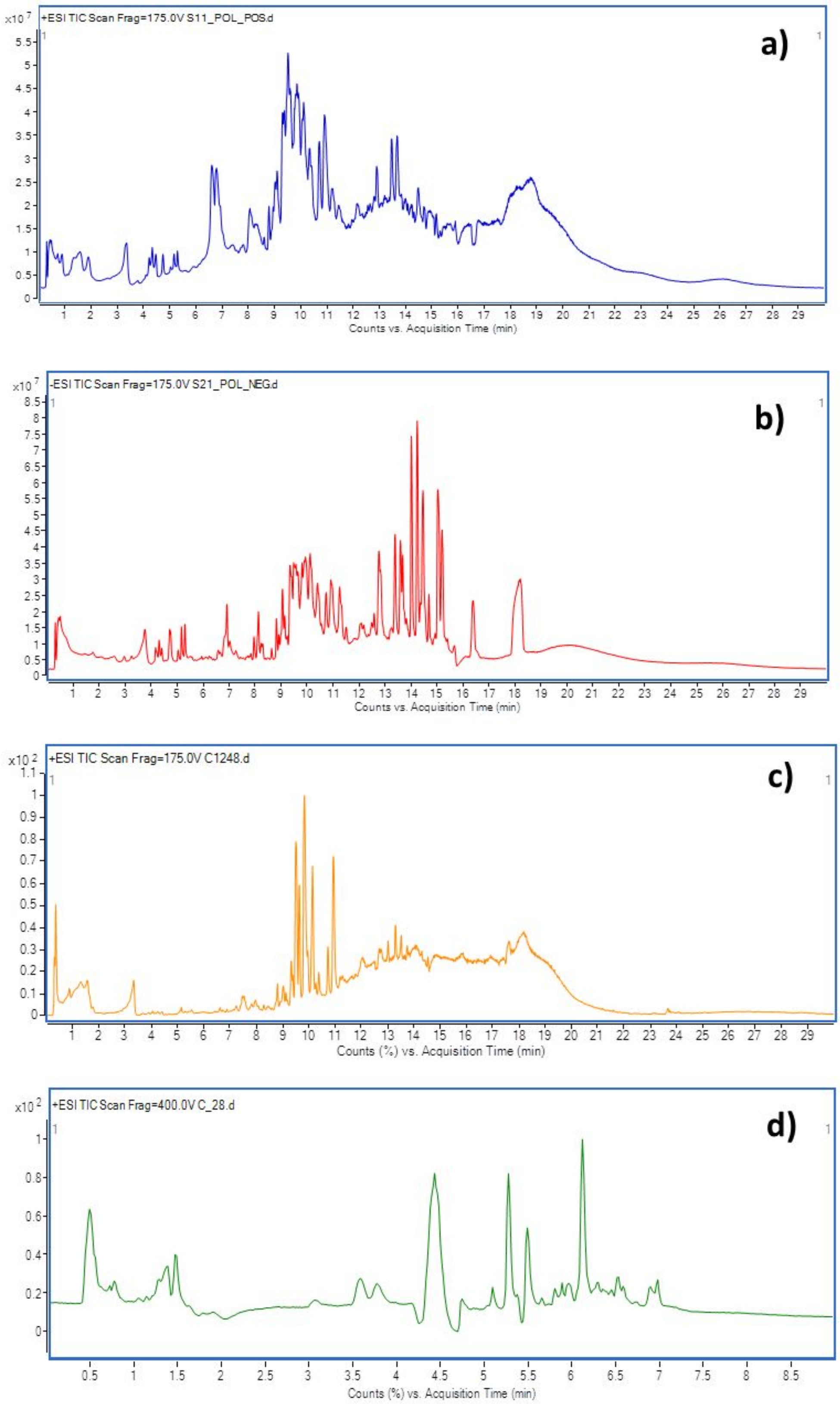

Figure S3. 2D-PLSDA plot pairwise PRE-CONTROL comparison of) aqueous extract determined by ESI(+)-RP-UHPLC-QTOF-MS; b) polar extract determined by ESI(-)-RP-UHPLC-QTOF-MS; c) organic extract determined by ESI(+)-RP-UHPLC-QTOF-MS; and d) polar extract determined by ESI(+)-HILIC-UHPLC-QTOF-MS. Black dots: CONTROL; green dots: PRE group.

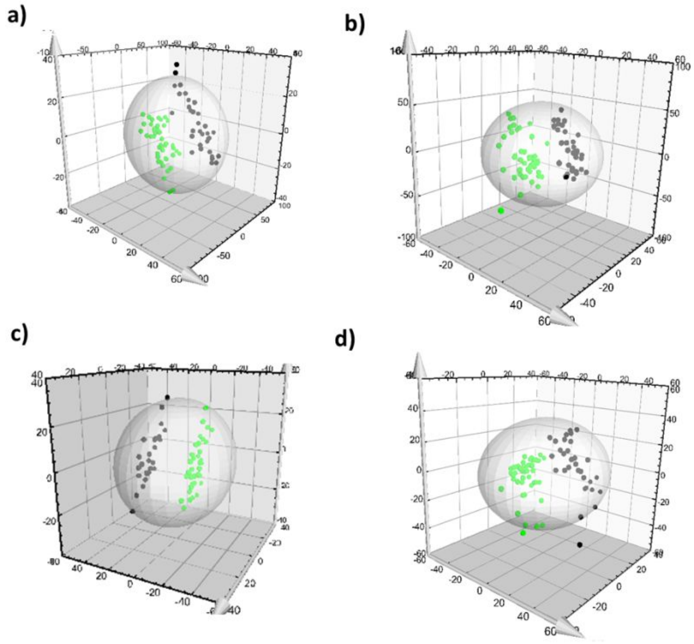

Figure S4. 2D-PLSDA plot pairwise POSTA-CONTROL comparison of) aqueous extract determined by ESI(+)-RP-UHPLC-QTOF-MS; b) polar extract determined by ESI(-)-RP-UHPLC-QTOF-MS; c) organic extract determined by ESI(+)-RP-UHPLC-QTOF-MS; and d) polar extract determined by ESI(+)-HILIC-UHPLC-QTOF-MS. Black dots: CONTROL; blue dots: POSTA group.

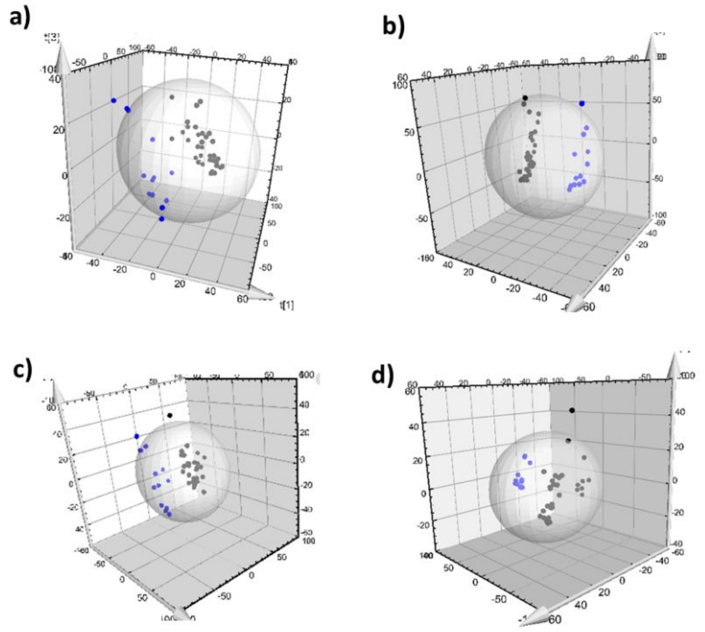

Figure S5. 2D-PLSDA plot pairwise POSTB-CONTROL comparison of) aqueous extract determined by ESI(+)-RP-UHPLC-QTOF-MS; b) polar extract determined by ESI(-)-RP-UHPLC-QTOF-MS; c) organic extract determined by ESI(+)-RP-UHPLC-QTOF-MS; and d) polar extract determined by ESI(+)-HILIC-UHPLC-QTOF-MS. Black dots: CONTROL; red dots: POSTB group.

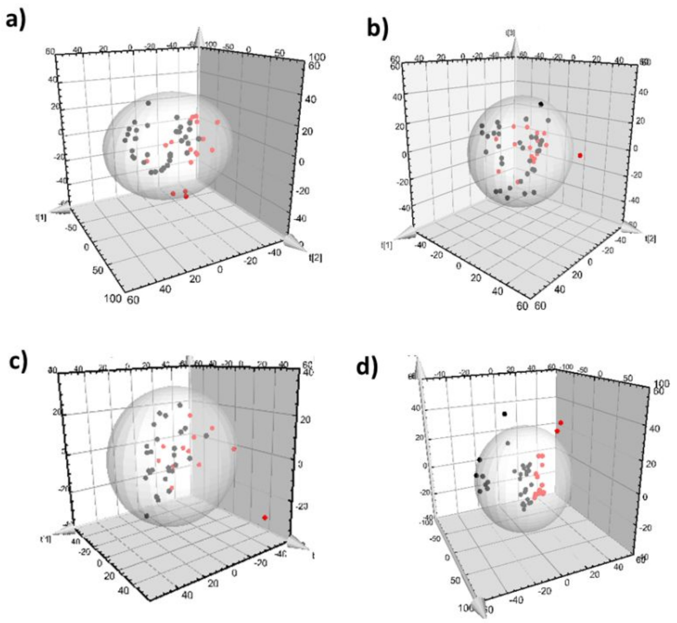

Figure S6. 2D-PLSDA plot pairwise PRE-POSTA comparison of) aqueous extract determined by ESI(+)-RP-UHPLC-QTOF-MS; b) polar extract determined by ESI(-)-RP-UHPLC-QTOF-MS; c) organic extract determined by ESI(+)-RP-UHPLC-QTOF-MS; and d) polar extract determined by ESI(+)-HILIC-UHPLC-QTOF-MS. Blue dots: POSTA group; green dots: PRE group.

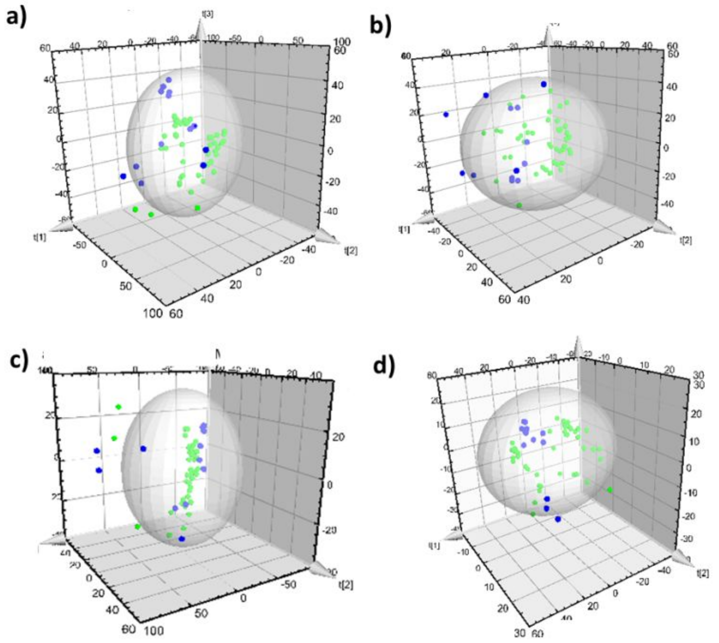

Figure S7. 2D-PLSDA plot pairwise PRE-POSTA comparison of) aqueous extract determined by ESI(+)-RP-UHPLC-QTOF-MS; b) polar extract determined by ESI(-)-RP-UHPLC-QTOF-MS; c) organic extract determined by ESI(+)-RP-UHPLC-QTOF-MS; and d) polar extract determined by ESI(+)-HILIC-UHPLC-QTOF-MS. Red dots: POSTB group; green dots: PRE group.

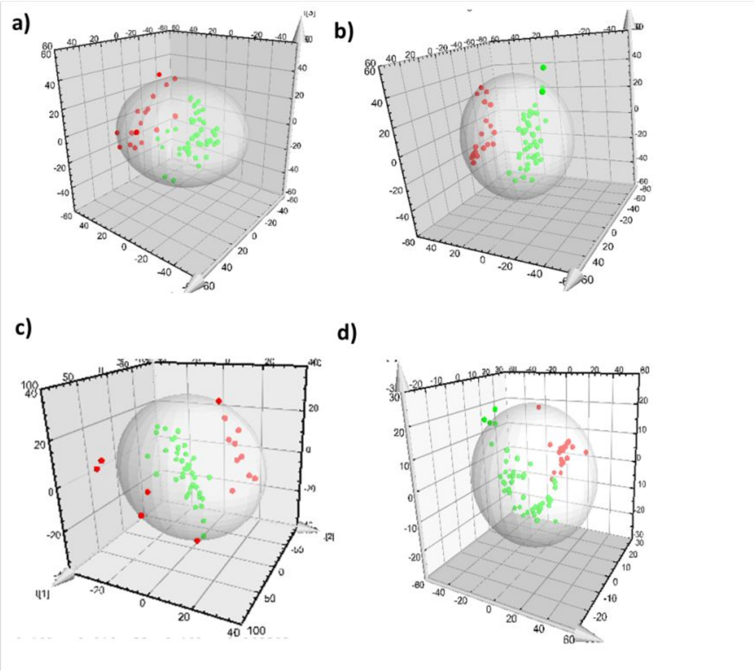

Figure S8. Main altered classes of metabolites in PRE, POSTA and POST B groups compared to CONTROL.

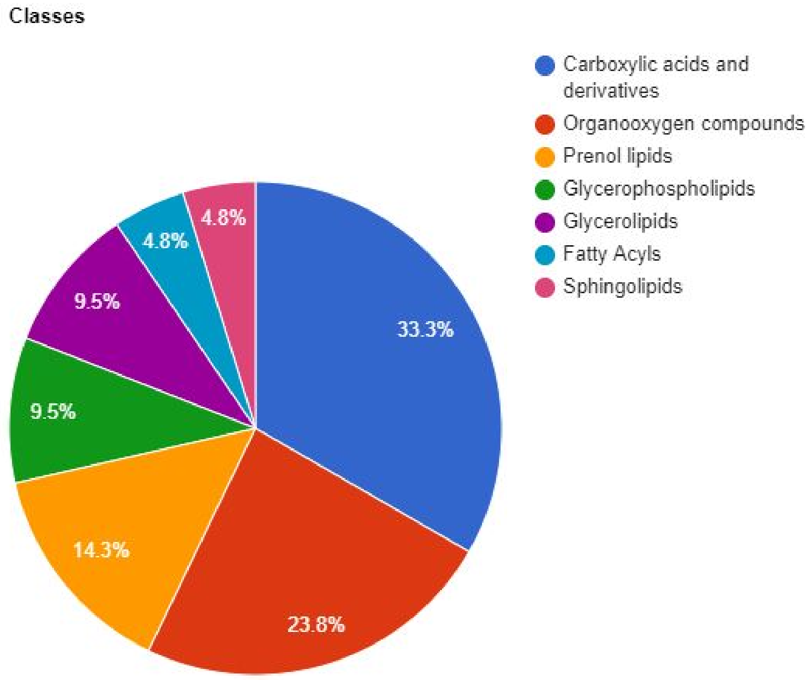

Supplement: Supplementary file 1 — pr3c00356_si_001.pdf [file pr3c00356_si_001.pdf]
